# Crystal structure of bis­(benzyl­amine-κ*N*)[5,10,15,20-tetra­kis­(4-chloro­phen­yl)porphyrinato-κ^4^ *N*]iron(II) *n*-hexane monosolvate

**DOI:** 10.1107/S2056989015024135

**Published:** 2016-01-01

**Authors:** Selma Dhifaoui, Wafa Harhouri, Anna Bujacz, Habib Nasri

**Affiliations:** a Laboratoire de Physico-chimie des Matériaux, Faculté des Sciences de Monastir, Avenue de l’environnement, 5019 Monastir, University of Monastir, Tunisia; b X-Ray Analysis Laboratory, Institute of Technical Biochemistry, Lodz University of Technology, Stefanowskiego 4/10, 90-924 Lodz, Poland

**Keywords:** crystal structure, hydrogen bonding, UV–vis spectra, iron(II)–porphyrin, benzyl­amine

## Abstract

In the title compound, [Fe^II^(C_44_H_24_Cl_4_N_4_)(C_6_H_5_CH_2_NH_2_)_2_]·C_6_H_14_ or [Fe^II^(TPP-Cl)(BzNH_2_)_2_]·*n*-hexane [where TPP-Cl and BzNH_2_ are 5,10,15,20-tetra­kis­(4-chloro­phen­yl)porphyrinate and benzyl­amine ligands, respectively], the Fe^II^ cation lies on an inversion centre and is octa­hedrally coordinated by the four pyrrole N atoms of the porphyrin ligand in the equatorial plane and by two amine N atoms of the benzyl­amine ligand in the axial sites. The crystal structure also contains one inversion-symmetric *n*-hexane solvent mol­ecule per complex mol­ecule. The average Fe—N_pyrrole_ bond length [1.994 (3) Å] indicates a low-spin complex. The crystal packing is sustained by N—H⋯Cl and C—H⋯Cl hydrogen-bonding inter­actions and by C—H⋯π inter­molecular inter­actions, leading to a three-dimensional network structure.

## Abstract 2

The crystal packing of the Fe^II^ porphyrin derivative results in channels parallel to [010] where the *n*-hexane solvent mol­ecules are located. UV–vis data of a CHCl_3_ solution and the solid of the title compound are also reported.

## Chemical context   

The structure of turnip cytochrome *f* has been determined on the basis of X-ray measurements (Martinez *et al.*, 1996 ▸), showing that the α-amino group of the Tyr-1 entity coordinates *trans* to the His-25 entity in the *c*-type heme protein. Thus, bis-amine Fe^II^ metalloporphyrins appear to be functionally significant as models for cytochrome *f*. On the other hand, it has been shown that the reaction of primary and secondary amines with iron(III) metalloporphyrins results in a base-catalysed one-electron reduction process and concomitant dissociation of the deprotonated amine radical (Del Gaudio & La Mar, 1978 ▸). It is also known that the addition of an excess of sterically unhindered alkylamines to an Fe(III) porphyrin derivative leads to bis­(amine)–iron(II) porphyrins with the central metal cation in a six-coordination (Morice *et al.*, 1998 ▸). Notably, the number of published structures of these type of iron(II) metalloporphyrins is small. In the Cambridge Structural Database (CSD, Version 5.35; Groom & Allen, 2014 ▸), only six amine porphyrin structures are reported, including [Fe^II^(TPP)(BzNH_2_)_2_] (TPP is the 5,10,15,20-tetra­phenyl­porphyrinato ligand; Bz is benz­yl) (Munro *et al.*, 1999 ▸).

We report herein the synthesis, the mol­ecular and crystal structures as well as UV-spectroscopic properties of bis(benzyl­amine)[5,10,15,20-tetra­(*para*-chloro­phen­yl)porphyrinato]iron(II) *n*-hexane monosolvate, [Fe^II^(TPP-Cl)(BzNH_2_)_2_])·*n*-hexane, (I).

## Structural commentary   

**Figure 1:**
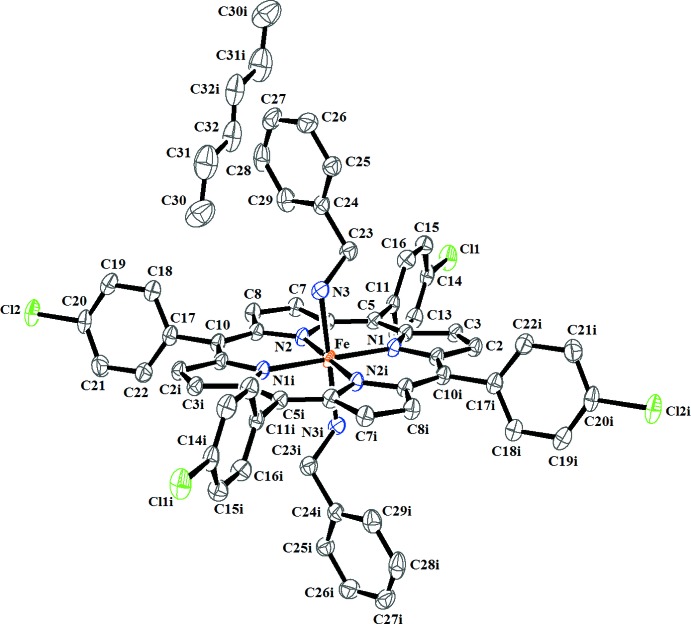
The structures of the mol­ecular entities in the title compound. Displacement ellipsoids are drawn at the 60% probability level. H atoms have been omitted for clarity. [Symmetry code: (i) −*x* + 1, −*y* + 1, −*z* + 1.]

The mol­ecular structure of (I) is illustrated in Fig. 1 ▸. The Fe^II^ cation is located on an inversion centre and shows an octa­hedral coordination environment. The equatorial plane is formed by the four nitro­gen atoms of the porphyrin moiety whereas the axial positions are occupied by the N atoms of the two benzyl­amine ligands.
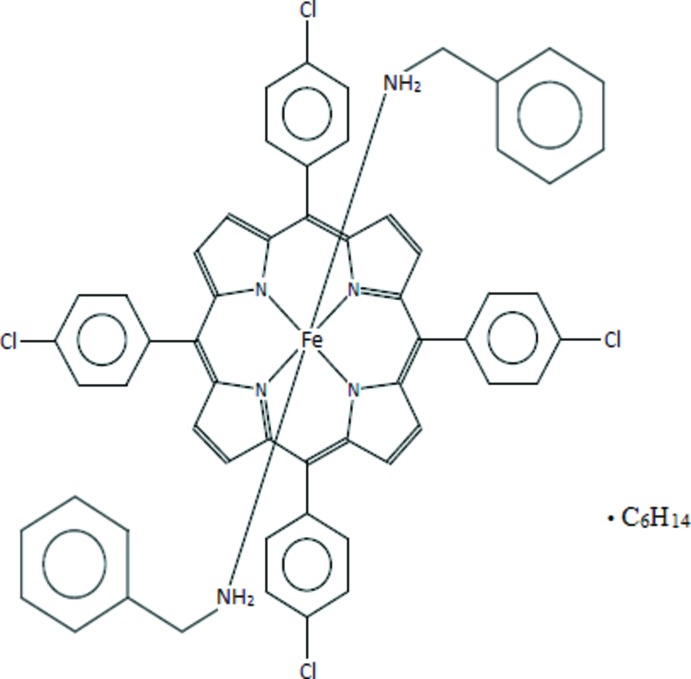


**Figure 2:**
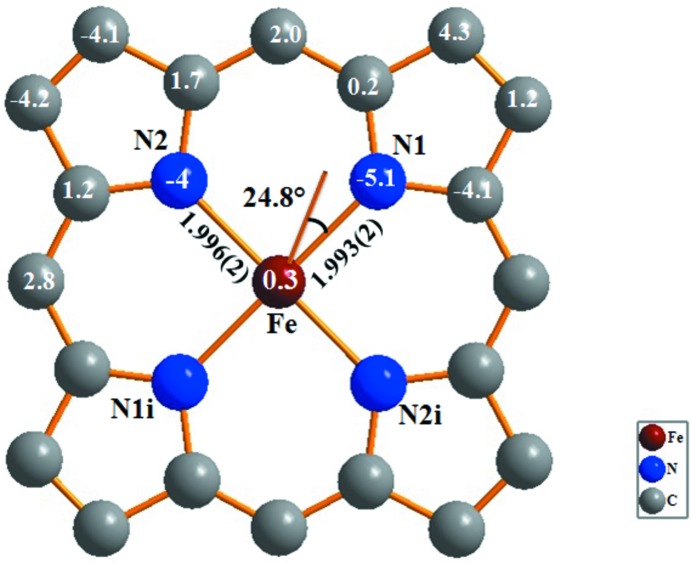
Schematic representation of the porphyrin core illustrating the displacements of each atom from the 24-atom plane in units of 0.01 Å.

The Fe—N_benzyl­amine_ bond length of 2.036 (2) Å is in the range of other iron(II)–bis­(amine) porphyrin complexes [1.799-2.285 Å] reported in the literature (CSD refcodes FAVGUE: Godbout *et al.*, 1999 ▸; IMELIV: Wyllie *et al.*, 2003 ▸) and is slightly smaller than in the related structure of [Fe^II^(TPP)(BzNH_2_)_2_] [2.043 (3) Å; Munro *et al.*, 1999 ▸]. The porphyrin core of (I) is represented in Fig. 2 ▸. The porphyrin macrocycle presents a nearly planar conformation with maximum and minimum deviations from the C_20_N_4_ least-squares plane of 0.044 (2) and −0.051 (2) Å for atoms C3 and N1, respectively, while the Fe^II^ cation is co-planar with this plane with a minute deviation of 0.003 (1) Å. The *α*-CH_2_ group of the benzyl­amine ligand is inclined at 24.8 (1)° relative to the shortest Fe—N_pyrrole_ bond (Fe—N1). This value is close to those of the related [Fe^II^(TPP)(BzNH_2_)_2_] derivative [18.2 (4), 30.1 (4)°; Munro *et al.*, 1999 ▸].

For iron(II) porphyrins, the relationship between the spin-state of the Fe^II^ cation and the value of the average equatorial Fe—N_pyrrole_ bond length has been discussed (Scheidt & Reed, 1981 ▸). For high-spin (*S* = 2) complexes, the Fe—N_pyrrole_ bond lengths are the longest, *e.g.* for the [Fe(TpivPP)(NO_3_)]^−^ complex (TpivPP = picket-fence porphyrin), Fe—N_pyrrole_ amounts to 2.070 (16) Å (Nasri *et al.*, 2006 ▸). For low-spin (*S* = 0) complexes, the average Fe—N_pyrrole_ bond length is shorter, *e.g.* for the [Fe(TPP)(4-MePip)_2_] complex (4-MePip is 4-methyl piperidine), the Fe—N_pyrrole_ bond length is 1.994 (4) Å (Munro & Ntshangase, 2003 ▸) and 1.990 (15) Å for the [Fe^II^(TpivPP)(NO_2_)(pyridine)]^−^ species (Nasri *et al.*, 2000 ▸). The inter­mediate spin state (*S* = 1) of Fe^II^ porphyrin complexes is represented by the shortest Fe—N_pyrrole_ distances, *e.g.* Fe(TTP) exhibits an Fe—N_pyrrole_ bond length of 1.979 (6) Å (Hu *et al.*, 2007 ▸). The averaged Fe—N_pyrrole_ bond length of 1.994 (3) Å for (I) is an indication that this species has a low-spin state (*S* = 0). This value is virtually the same as in the related [Fe^II^(TPP)(BzNH_2_)_2_] derivative [Fe—N_pyrrole_ = 1.992 (4) Å; Munro *et al.*, 1999 ▸].

## Supra­molecular features   

**Figure 3:**
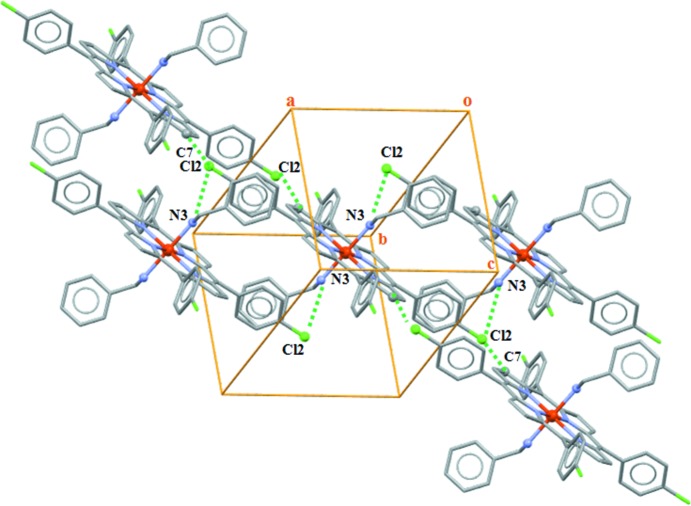
A partial view of the crystal packing of (I), showing the linkage between the [Fe^II^(TPP-Cl)(BzNH_2_)_2_] complexes through C—H⋯Cl and N—H⋯Cl hydrogen bonds. The *n*-hexane solvent mol­ecules have been omitted for clarity.

The complex mol­ecules are packed in such a way that channels are formed parallel to [010] in which the *n*-hexane mol­ecules are situated. The linkage of the mol­ecular components in the crystal structure of (I) is accomplished by C—H⋯Cl, N—H⋯Cl hydrogen-bonding inter­actions as well as C—H⋯π inter­actions (Figs. 3 ▸ and 4 ▸; Table 1 ▸). Each [Fe^II^(TPP-Cl)(BzNH_2_)_2_] complex is linked to neighbouring complexes through N—H⋯Cl hydrogen bonds between the N3 atom of the benzyl­amine ligand and the Cl2 atom of a TPP-Cl moiety and by C—H⋯Cl inter­actions between the pyrrole C7 atom and the Cl2 atom. In addition, the phenyl C19 atom of the [Fe^II^(TClPP)(BzNH_2_)_2_] complex inter­acts with the centroid *Cg*1 of the (N1/C1–C4) pyrrole ring through C—H⋯π inter­actions. The three-dimensional supra­molecular network is consolidated by another C—H⋯π intra­molecular inter­action involving the C31 atom of the *n*-hexane solvent mol­ecule and the centroid *Cg*7 of the (C11–C16) phenyl ring.

## Synthesis   

### Synthesis of 5,10,15,20-tetra­(*para*-chloro­phen­yl)porph­yrin   

In a 100 ml two-necked flask, 4-chloro­benzaldehyde (6 g, 42 mmol) was dissolved in 50 ml of propionic acid. The solution was heated under reflex at 413 K. Freshly distilled pyrrole (3.36 ml, 42 mmol) was added dropwise and the mixture stirred for another 40 min. The mixture was then cooled overnight to 277 K and filtered *in vacuo*. The crude product was purified using column chromatography (chloro­form/hexane = 4/1 *v*/*v* as an eluent). A purple solid was obtained that was dried *in vacuo* (1.5 g, yield 25%). UV–vis spectrum in CHCl_3_: λ_max_ (10^−3^·∊) 420 (512.7), 516 (16.7), 552 (7.4), 591 (4.7), 646 (4.0).

### Metallation of the porphyrin and synthesis of (triflato)[5,10,15,20-tetra­(*para*-chloro­phen­yl)porphyrin­ato]iron(III)   

The metallation of the porphyrin was performed using the literature method to yield the chlorido–iron(III) derivative [Fe^III^(TPP-Cl)Cl] (Collman *et al.*, 1975 ▸). We used the triflato–iron(III) TPP-Cl derivative [Fe^III^(TPP-Cl)(SO_3_CF_3_)] as starting material because the triflato ligand (SO_3_CF_3_^−^) is much easier to substitute than the chlorido ligand. This complex was prepared according to a literature protocol (Gismelseed *et al.*, 1990 ▸).

### Synthesis and crystallization of bis­(benzyl­amine-κ*N*)[5,10,15,20-tetra­kis­(4-chloro­phen­yl)porphyrinato-κ^4^ *N*]iron(II) *n*-hexane monosolvate complex, (I)   

To a solution of [Fe^III^(TPP-Cl)(SO_3_CF_3_)] (Gismelseed *et al.*, 1990 ▸) (15 mg, 0.0156 mmol) in di­chloro­methane (15 ml) was added an excess of benzyl­amine (50 mg, 0.48 mmol). The reaction mixture was stirred at room temperature for 2 h. Crystals of the title complex were obtained by diffusion of *n*-hexane through the di­chloro­methane solution.

## UV–vis spectra   

**Figure 5:**
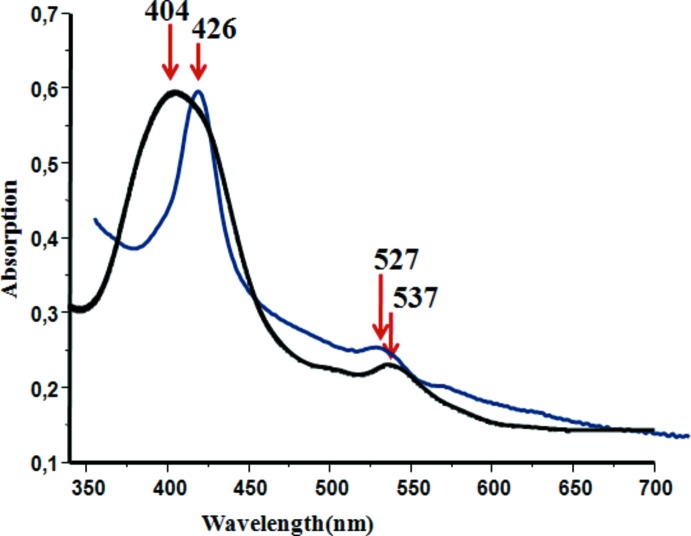
UV–vis spectra of the solid [Fe^III^(TClPP)(SO_3_CF_3_)] starting material (black) and solid (I) (blue).

The UV–visible spectra with absorption bands at λ_max_ 425/426, 532/527, 562/566 nm (CHCl_3_ solution/solid state) were recorded on a WinASPECT PLUS (validation for SPECORD PLUS version 4.2) scanning spectrophotometer. In Fig. 5 ▸ are illustrated the electronic spectra of the solid [Fe^III^(TPP-Cl)(SO_3_CF_3_)] complex, used as starting material, and complex (I) which shows that the Soret band of the latter species is red-shifted compared to the one of the starting material. The λ_max_ values of the Soret and *Q* bands of (I) in the solid state and in chloro­form solution are very close. These values also compare well with those of the related [Fe^II^(TPP)(*L*)_2_] (*L* = 1-BuNH_2_, BzNH_2_, PhCH_2_CH_2_NH_2_) species (Munro *et al.*, 1999 ▸).

## Refinement   

Crystal data, data collection and structure refinement details are summarized in Table 2 ▸. H atoms were positioned geometrically and refined using a riding model with C—H = 0.93 Å (aromatic), 0.97 Å (methyl­ene), 0.96 Å (meth­yl) and N—H = 0.89 Å for the axial ligand, with *U_iso_*(H_phen­yl_, H_methyl­ene_, H_amine_) = 1.2*U*_eq_(C/N) and *U*_iso_(H_meth­yl_) = 1.5*U_eq_*(C).

## Supplementary Material

Crystal structure: contains datablock(s) I. DOI: 10.1107/S2056989015024135/wm5253sup1.cif

Structure factors: contains datablock(s) I. DOI: 10.1107/S2056989015024135/wm5253Isup2.hkl

CCDC reference: 1442707

Additional supporting information:  crystallographic information; 3D view; checkCIF report

## Figures and Tables

**Figure 4:**
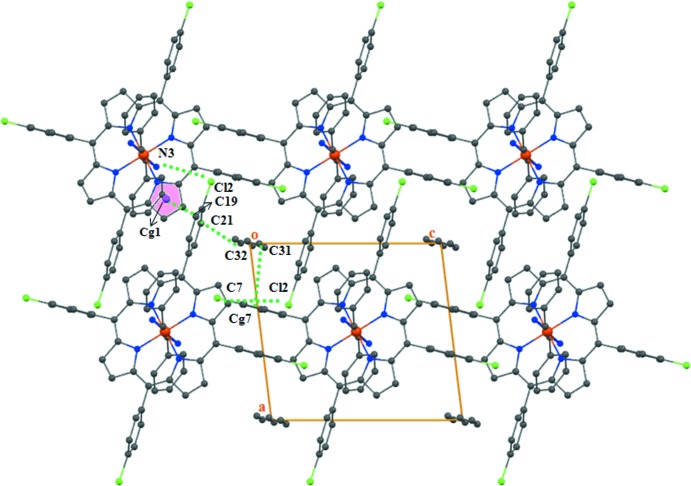
The crystal structure of the title compound plotted in a projection along [010]. Contacts between the entities are given as dashed lines.

**Table 1:** Hydrogen-bond geometry (Å, °) *Cg*1 and *Cg*7 are the centroids of the N1/C1–C4 and C11–C16 rings, respectively.

*D*—H⋯*A*	*D*—H	H⋯*A*	*D*⋯*A*	*D*—H⋯*A*
C19—H19⋯*Cg*1^i^	0.93	2.66	3.586 (3)	133
C31—H31*A*⋯*Cg*7^i^	0.97	2.63	3.701 (5)	160
N3—H3*B*⋯Cl2^ii^	0.89	2.68	3.651 (2)	133
C7—H7⋯Cl2^iii^	0.93	3.00	3.926 (2)	175

Symmetry codes: (i) 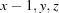; (ii) 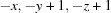; (iii) 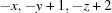.

**Table 2:** Experimental details

Crystal data	
Chemical formula	[Fe(C_44_H_24_Cl_4_N_4_)(C_7_H_9_N)_2_]·C_6_H_14_
*M*_r_	1106.79
Crystal system, space group	Triclinic, *P* 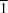
Temperature (K)	100
*a*, *b*, *c* (Å)	10.7986 (6), 11.0555 (6), 11.4118 (4)
α, β, γ (°)	87.918 (4), 82.785 (4), 79.815 (5)
*V* (Å^3^)	1330.16 (12)
*Z*	1
Radiation type	Cu *K*α
μ (mm^−1^)	4.50
Crystal size (mm)	0.4 × 0.3 × 0.1

Data collection	
Diffractometer	Agilent SuperNova Dual Source diffractometer with a TitanS2 detector
Absorption correction	Multi-scan (*CrysAlis PRO*; Agilent, 2014 ▸)
*T*_min_, *T*_max_	0.416, 1.000
No. of measured, independent and observed [*I* > 2σ(*I*)] reflections	12765, 5355, 4825
*R*_int_	0.028
(sin θ/λ)_max_ (Å^−1^)	0.627

Refinement	
*R*[*F* ^2^ > 2σ(*F* ^2^)], *wR*(*F* ^2^), *S*	0.048, 0.133, 1.06
No. of reflections	5355
No. of parameters	340
H-atom treatment	H-atom parameters constrained
Δρ_max_, Δρ_min_ (e Å^−3^)	1.09, −0.54

Computer programs: *CrysAlis PRO* (Agilent, 2014 ▸), *SIR2004* (Burla *et al.*, 2005 ▸), *SHELXL2014* (Sheldrick, 2015 ▸), *ORTEPIII* (Burnett & Johnson, 1996 ▸) and *ORTEP-3 for Windows* and *WinGX* (Farrugia, 2012 ▸).

## supplementary crystallographic information

### Crystal data

**Table:**

[Fe(C_44_H_24_Cl_4_N_4_)(C_7_H_9_N)_2_]·C_6_H_14_	*Z* = 1
*M**_r_* = 1106.79	*F*(000) = 576
Triclinic, *P*1	*D*_x_ = 1.382 Mg m^−^^3^
*a* = 10.7986 (6) Å	Cu *K*α radiation, λ = 1.54184 Å
*b* = 11.0555 (6) Å	Cell parameters from 10827 reflections
*c* = 11.4118 (4) Å	θ = 4.1–74.9°
α = 87.918 (4)°	µ = 4.50 mm^−^^1^
β = 82.785 (4)°	*T* = 100 K
γ = 79.815 (5)°	Prism, dark red
*V* = 1330.16 (12) Å^3^	0.4 × 0.3 × 0.1 mm

### Data collection

**Table:**

Agilent SuperNova Dual Source diffractometer with a TitanS2 detector	5355 independent reflections
Radiation source: sealed X-ray tube	4825 reflections with *I* > 2σ(*I*)
Detector resolution: 4.1685 pixels mm^-1^	*R*_int_ = 0.028
ω scans	θ_max_ = 75.3°, θ_min_ = 3.9°
Absorption correction: multi-scan (*CrysAlis PRO*; Agilent, 2014)	*h* = −13→13
*T*_min_ = 0.416, *T*_max_ = 1.000	*k* = −13→10
12765 measured reflections	*l* = −14→12

### Refinement

**Table:**

Refinement on *F*^2^	0 restraints
Least-squares matrix: full	Hydrogen site location: inferred from neighbouring sites
*R*[*F*^2^ > 2σ(*F*^2^)] = 0.048	H-atom parameters constrained
*wR*(*F*^2^) = 0.133	*w* = 1/[σ^2^(*F*_o_^2^) + (0.0771*P*)^2^ + 0.8782*P*] where *P* = (*F*_o_^2^ + 2*F*_c_^2^)/3
*S* = 1.06	(Δ/σ)_max_ = 0.001
5355 reflections	Δρ_max_ = 1.09 e Å^−^^3^
340 parameters	Δρ_min_ = −0.53 e Å^−^^3^

### Special details

**Table:**

Geometry. All e.s.d.'s (except the e.s.d. in the dihedral angle between two l.s. planes) are estimated using the full covariance matrix. The cell e.s.d.'s are taken into account individually in the estimation of e.s.d.'s in distances, angles and torsion angles; correlations between e.s.d.'s in cell parameters are only used when they are defined by crystal symmetry. An approximate (isotropic) treatment of cell e.s.d.'s is used for estimating e.s.d.'s involving l.s. planes.

### Fractional atomic coordinates and isotropic or equivalent isotropic displacement parameters (Å^2^)

**Table:**

	*x*	*y*	*z*	*U*_iso_*/*U*_eq_	
Fe	0.5000	0.5000	0.5000	0.01855 (14)	
N1	0.65294 (17)	0.39615 (18)	0.55478 (16)	0.0209 (4)	
N2	0.40204 (17)	0.47595 (18)	0.65683 (16)	0.0210 (4)	
N3	0.43283 (18)	0.35548 (18)	0.44078 (17)	0.0238 (4)	
H3A	0.3486	0.3738	0.4512	0.029*	
H3B	0.4563	0.3509	0.3632	0.029*	
C1	0.7729 (2)	0.3693 (2)	0.4932 (2)	0.0224 (4)	
C2	0.8596 (2)	0.2991 (2)	0.5658 (2)	0.0242 (5)	
H2	0.9452	0.2700	0.5437	0.029*	
C3	0.7939 (2)	0.2831 (2)	0.6725 (2)	0.0242 (5)	
H3	0.8255	0.2414	0.7377	0.029*	
C4	0.6649 (2)	0.3434 (2)	0.6655 (2)	0.0221 (4)	
C5	0.5680 (2)	0.3471 (2)	0.75867 (19)	0.0219 (4)	
C6	0.4447 (2)	0.4080 (2)	0.75297 (19)	0.0226 (5)	
C7	0.3420 (2)	0.4066 (2)	0.8463 (2)	0.0250 (5)	
H7	0.3468	0.3666	0.9191	0.030*	
C8	0.2375 (2)	0.4742 (2)	0.8080 (2)	0.0254 (5)	
H8	0.1568	0.4897	0.8497	0.030*	
C9	0.2745 (2)	0.5176 (2)	0.6907 (2)	0.0225 (4)	
C10	0.1926 (2)	0.5909 (2)	0.6214 (2)	0.0232 (5)	
C11	0.59738 (19)	0.2788 (2)	0.87045 (19)	0.0220 (5)	
C12	0.6263 (2)	0.3395 (2)	0.9655 (2)	0.0269 (5)	
H12	0.6267	0.4236	0.9597	0.032*	
C13	0.6548 (2)	0.2760 (3)	1.0695 (2)	0.0295 (5)	
H13	0.6735	0.3172	1.1329	0.035*	
C14	0.6547 (2)	0.1513 (3)	1.0766 (2)	0.0281 (5)	
C15	0.6267 (2)	0.0882 (2)	0.9836 (2)	0.0302 (5)	
H15	0.6276	0.0039	0.9895	0.036*	
C16	0.5972 (2)	0.1531 (2)	0.8810 (2)	0.0289 (5)	
H16	0.5770	0.1117	0.8185	0.035*	
C17	0.0571 (2)	0.6294 (2)	0.6735 (2)	0.0252 (5)	
C18	−0.0257 (2)	0.5459 (3)	0.6854 (2)	0.0306 (5)	
H18	0.0029	0.4657	0.6600	0.037*	
C19	−0.1513 (2)	0.5807 (3)	0.7349 (2)	0.0329 (6)	
H19	−0.2063	0.5242	0.7431	0.039*	
C20	−0.1927 (2)	0.6994 (3)	0.7714 (2)	0.0296 (5)	
C21	−0.1134 (3)	0.7858 (3)	0.7565 (3)	0.0384 (6)	
H21	−0.1433	0.8668	0.7788	0.046*	
C22	0.0120 (2)	0.7492 (3)	0.7077 (3)	0.0352 (6)	
H22	0.0662	0.8065	0.6980	0.042*	
C23	0.4696 (2)	0.2302 (2)	0.4923 (2)	0.0276 (5)	
H23A	0.4913	0.2379	0.5713	0.033*	
H23B	0.5444	0.1877	0.4449	0.033*	
C24	0.3655 (2)	0.1546 (2)	0.4982 (2)	0.0258 (5)	
C25	0.3711 (2)	0.0611 (2)	0.4198 (2)	0.0304 (5)	
H25	0.4395	0.0450	0.3611	0.036*	
C26	0.2759 (3)	−0.0093 (3)	0.4274 (3)	0.0397 (6)	
H26	0.2810	−0.0722	0.3741	0.048*	
C27	0.1738 (3)	0.0136 (3)	0.5138 (3)	0.0423 (7)	
H27	0.1102	−0.0339	0.5191	0.051*	
C28	0.1663 (3)	0.1070 (3)	0.5921 (3)	0.0428 (7)	
H28	0.0971	0.1230	0.6500	0.051*	
C29	0.2623 (3)	0.1784 (3)	0.5854 (2)	0.0347 (6)	
H29	0.2570	0.2412	0.6389	0.042*	
Cl1	0.69094 (6)	0.07092 (7)	1.20562 (5)	0.03984 (18)	
Cl2	−0.34861 (5)	0.74242 (7)	0.83650 (5)	0.03639 (17)	
C30	−0.0017 (4)	0.2924 (5)	0.9519 (4)	0.0738 (12)	
H30A	−0.0290	0.3479	0.8900	0.111*	
H30B	−0.0494	0.3193	1.0259	0.111*	
H30C	0.0868	0.2909	0.9562	0.111*	
C31	−0.0232 (4)	0.1616 (5)	0.9258 (4)	0.0739 (13)	
H31A	−0.1125	0.1668	0.9185	0.089*	
H31B	0.0230	0.1384	0.8492	0.089*	
C32	0.0130 (3)	0.0575 (4)	1.0118 (3)	0.0581 (9)	
H32A	0.1032	0.0489	1.0161	0.070*	
H32B	−0.0301	0.0817	1.0893	0.070*	

### Atomic displacement parameters (Å^2^)

**Table:**

	*U*^11^	*U*^22^	*U*^33^	*U*^12^	*U*^13^	*U*^23^
Fe	0.0152 (2)	0.0252 (3)	0.0155 (2)	−0.00339 (18)	−0.00488 (17)	0.00556 (18)
N1	0.0167 (8)	0.0284 (10)	0.0176 (9)	−0.0040 (7)	−0.0038 (7)	0.0055 (7)
N2	0.0171 (8)	0.0276 (10)	0.0181 (9)	−0.0025 (7)	−0.0056 (7)	0.0058 (7)
N3	0.0236 (9)	0.0278 (10)	0.0217 (9)	−0.0068 (8)	−0.0083 (7)	0.0063 (7)
C1	0.0178 (10)	0.0269 (11)	0.0231 (11)	−0.0038 (8)	−0.0067 (8)	0.0056 (9)
C2	0.0174 (10)	0.0299 (12)	0.0252 (11)	−0.0028 (8)	−0.0062 (8)	0.0058 (9)
C3	0.0208 (11)	0.0291 (12)	0.0239 (11)	−0.0046 (9)	−0.0095 (8)	0.0080 (9)
C4	0.0208 (10)	0.0268 (11)	0.0198 (10)	−0.0046 (8)	−0.0075 (8)	0.0053 (8)
C5	0.0221 (10)	0.0269 (11)	0.0179 (10)	−0.0055 (8)	−0.0073 (8)	0.0055 (8)
C6	0.0228 (11)	0.0281 (12)	0.0172 (10)	−0.0046 (9)	−0.0046 (8)	0.0049 (8)
C7	0.0242 (11)	0.0316 (12)	0.0185 (10)	−0.0035 (9)	−0.0040 (8)	0.0073 (9)
C8	0.0210 (10)	0.0336 (13)	0.0200 (11)	−0.0027 (9)	−0.0010 (8)	0.0051 (9)
C9	0.0199 (10)	0.0277 (11)	0.0198 (10)	−0.0041 (8)	−0.0033 (8)	0.0041 (8)
C10	0.0178 (10)	0.0290 (12)	0.0224 (11)	−0.0029 (8)	−0.0038 (8)	0.0035 (9)
C11	0.0160 (10)	0.0312 (12)	0.0183 (10)	−0.0021 (8)	−0.0044 (8)	0.0068 (9)
C12	0.0244 (11)	0.0342 (13)	0.0220 (11)	−0.0047 (9)	−0.0051 (9)	0.0052 (9)
C13	0.0262 (11)	0.0444 (15)	0.0184 (11)	−0.0054 (10)	−0.0068 (9)	0.0037 (10)
C14	0.0177 (10)	0.0451 (14)	0.0197 (11)	−0.0021 (9)	−0.0038 (8)	0.0121 (10)
C15	0.0294 (12)	0.0327 (13)	0.0277 (12)	−0.0039 (10)	−0.0050 (10)	0.0100 (10)
C16	0.0305 (12)	0.0348 (13)	0.0224 (11)	−0.0069 (10)	−0.0072 (9)	0.0064 (10)
C17	0.0201 (11)	0.0347 (13)	0.0201 (11)	−0.0028 (9)	−0.0049 (8)	0.0087 (9)
C18	0.0225 (11)	0.0369 (14)	0.0318 (13)	−0.0038 (10)	−0.0030 (9)	0.0001 (10)
C19	0.0214 (11)	0.0459 (15)	0.0322 (13)	−0.0093 (10)	−0.0038 (10)	0.0050 (11)
C20	0.0166 (10)	0.0465 (15)	0.0229 (11)	0.0009 (10)	−0.0026 (8)	0.0079 (10)
C21	0.0279 (13)	0.0364 (15)	0.0464 (16)	0.0011 (11)	0.0021 (11)	0.0017 (12)
C22	0.0232 (12)	0.0342 (14)	0.0474 (16)	−0.0051 (10)	−0.0024 (11)	0.0060 (11)
C23	0.0253 (11)	0.0303 (12)	0.0285 (12)	−0.0045 (9)	−0.0104 (9)	0.0049 (9)
C24	0.0231 (11)	0.0284 (12)	0.0262 (11)	−0.0027 (9)	−0.0095 (9)	0.0102 (9)
C25	0.0273 (12)	0.0308 (13)	0.0336 (13)	−0.0052 (10)	−0.0072 (10)	0.0050 (10)
C26	0.0365 (14)	0.0335 (14)	0.0526 (17)	−0.0088 (11)	−0.0167 (13)	0.0054 (12)
C27	0.0281 (13)	0.0420 (16)	0.0606 (19)	−0.0124 (11)	−0.0183 (13)	0.0248 (14)
C28	0.0233 (12)	0.0570 (19)	0.0416 (15)	0.0028 (11)	−0.0005 (11)	0.0270 (14)
C29	0.0326 (13)	0.0393 (14)	0.0289 (13)	0.0012 (11)	−0.0035 (10)	0.0085 (11)
Cl1	0.0326 (3)	0.0590 (4)	0.0244 (3)	−0.0004 (3)	−0.0064 (2)	0.0208 (3)
Cl2	0.0187 (3)	0.0606 (4)	0.0256 (3)	0.0012 (2)	0.0002 (2)	0.0068 (3)
C30	0.070 (3)	0.086 (3)	0.074 (3)	−0.035 (2)	−0.018 (2)	0.021 (2)
C31	0.053 (2)	0.105 (4)	0.060 (2)	−0.003 (2)	−0.0111 (18)	0.014 (2)
C32	0.0321 (15)	0.088 (3)	0.0495 (19)	0.0010 (16)	−0.0071 (14)	0.0095 (19)

### Geometric parameters (Å, º)

**Table:**

Fe—N1	1.9932 (18)	C15—C16	1.393 (3)
Fe—N1^i^	1.9932 (18)	C15—H15	0.9300
Fe—N2	1.9955 (18)	C16—H16	0.9300
Fe—N2^i^	1.9956 (18)	C17—C22	1.381 (4)
Fe—N3^i^	2.036 (2)	C17—C18	1.386 (4)
Fe—N3	2.036 (2)	C18—C19	1.396 (3)
N1—C1	1.382 (3)	C18—H18	0.9300
N1—C4	1.384 (3)	C19—C20	1.371 (4)
N2—C9	1.383 (3)	C19—H19	0.9300
N2—C6	1.387 (3)	C20—C21	1.384 (4)
N3—C23	1.492 (3)	C20—Cl2	1.744 (2)
N3—H3A	0.8900	C21—C22	1.393 (4)
N3—H3B	0.8900	C21—H21	0.9300
C1—C10^i^	1.391 (3)	C22—H22	0.9300
C1—C2	1.435 (3)	C23—C24	1.509 (3)
C2—C3	1.353 (3)	C23—H23A	0.9700
C2—H2	0.9300	C23—H23B	0.9700
C3—C4	1.444 (3)	C24—C25	1.380 (4)
C3—H3	0.9300	C24—C29	1.392 (4)
C4—C5	1.391 (3)	C25—C26	1.388 (4)
C5—C6	1.390 (3)	C25—H25	0.9300
C5—C11	1.498 (3)	C26—C27	1.377 (5)
C6—C7	1.439 (3)	C26—H26	0.9300
C7—C8	1.351 (3)	C27—C28	1.375 (5)
C7—H7	0.9300	C27—H27	0.9300
C8—C9	1.438 (3)	C28—C29	1.403 (4)
C8—H8	0.9300	C28—H28	0.9300
C9—C10	1.393 (3)	C29—H29	0.9300
C10—C1^i^	1.391 (3)	C30—C31	1.550 (7)
C10—C17	1.502 (3)	C30—H30A	0.9600
C11—C16	1.391 (4)	C30—H30B	0.9600
C11—C12	1.392 (3)	C30—H30C	0.9600
C12—C13	1.396 (3)	C31—C32	1.514 (6)
C12—H12	0.9300	C31—H31A	0.9700
C13—C14	1.378 (4)	C31—H31B	0.9700
C13—H13	0.9300	C32—C32^ii^	1.392 (9)
C14—C15	1.384 (4)	C32—H32A	0.9700
C14—Cl1	1.742 (2)	C32—H32B	0.9700
			
N1—Fe—N1^i^	180.0	C15—C14—Cl1	119.1 (2)
N1—Fe—N2	89.69 (8)	C14—C15—C16	118.8 (2)
N1^i^—Fe—N2	90.31 (8)	C14—C15—H15	120.6
N1—Fe—N2^i^	90.31 (8)	C16—C15—H15	120.6
N1^i^—Fe—N2^i^	89.69 (8)	C11—C16—C15	121.2 (2)
N2—Fe—N2^i^	180.0	C11—C16—H16	119.4
N1—Fe—N3^i^	85.61 (8)	C15—C16—H16	119.4
N1^i^—Fe—N3^i^	94.39 (8)	C22—C17—C18	118.9 (2)
N2—Fe—N3^i^	92.04 (8)	C22—C17—C10	120.6 (2)
N2^i^—Fe—N3^i^	87.96 (8)	C18—C17—C10	120.5 (2)
N1—Fe—N3	94.39 (8)	C17—C18—C19	120.8 (3)
N1^i^—Fe—N3	85.61 (8)	C17—C18—H18	119.6
N2—Fe—N3	87.96 (8)	C19—C18—H18	119.6
N2^i^—Fe—N3	92.04 (8)	C20—C19—C18	119.1 (2)
N3^i^—Fe—N3	180.0	C20—C19—H19	120.4
C1—N1—C4	104.92 (18)	C18—C19—H19	120.4
C1—N1—Fe	127.30 (15)	C19—C20—C21	121.2 (2)
C4—N1—Fe	127.61 (15)	C19—C20—Cl2	119.3 (2)
C9—N2—C6	104.94 (18)	C21—C20—Cl2	119.5 (2)
C9—N2—Fe	127.24 (15)	C20—C21—C22	118.8 (3)
C6—N2—Fe	127.72 (15)	C20—C21—H21	120.6
C23—N3—Fe	119.69 (15)	C22—C21—H21	120.6
C23—N3—H3A	107.4	C17—C22—C21	121.1 (3)
Fe—N3—H3A	107.4	C17—C22—H22	119.5
C23—N3—H3B	107.4	C21—C22—H22	119.5
Fe—N3—H3B	107.4	N3—C23—C24	112.64 (19)
H3A—N3—H3B	106.9	N3—C23—H23A	109.1
N1—C1—C10^i^	125.2 (2)	C24—C23—H23A	109.1
N1—C1—C2	110.57 (19)	N3—C23—H23B	109.1
C10^i^—C1—C2	124.1 (2)	C24—C23—H23B	109.1
C3—C2—C1	107.4 (2)	H23A—C23—H23B	107.8
C3—C2—H2	126.3	C25—C24—C29	119.2 (2)
C1—C2—H2	126.3	C25—C24—C23	121.4 (2)
C2—C3—C4	106.6 (2)	C29—C24—C23	119.4 (2)
C2—C3—H3	126.7	C24—C25—C26	120.9 (3)
C4—C3—H3	126.7	C24—C25—H25	119.6
N1—C4—C5	125.7 (2)	C26—C25—H25	119.6
N1—C4—C3	110.55 (19)	C27—C26—C25	120.2 (3)
C5—C4—C3	123.7 (2)	C27—C26—H26	119.9
C6—C5—C4	123.7 (2)	C25—C26—H26	119.9
C6—C5—C11	118.1 (2)	C28—C27—C26	119.6 (3)
C4—C5—C11	118.13 (19)	C28—C27—H27	120.2
N2—C6—C5	125.4 (2)	C26—C27—H27	120.2
N2—C6—C7	110.37 (19)	C27—C28—C29	120.6 (3)
C5—C6—C7	124.2 (2)	C27—C28—H28	119.7
C8—C7—C6	107.1 (2)	C29—C28—H28	119.7
C8—C7—H7	126.5	C24—C29—C28	119.5 (3)
C6—C7—H7	126.5	C24—C29—H29	120.3
C7—C8—C9	107.2 (2)	C28—C29—H29	120.3
C7—C8—H8	126.4	C31—C30—H30A	109.5
C9—C8—H8	126.4	C31—C30—H30B	109.5
N2—C9—C10	125.1 (2)	H30A—C30—H30B	109.5
N2—C9—C8	110.46 (19)	C31—C30—H30C	109.5
C10—C9—C8	124.4 (2)	H30A—C30—H30C	109.5
C1^i^—C10—C9	124.7 (2)	H30B—C30—H30C	109.5
C1^i^—C10—C17	117.5 (2)	C32—C31—C30	119.2 (4)
C9—C10—C17	117.8 (2)	C32—C31—H31A	107.5
C16—C11—C12	118.6 (2)	C30—C31—H31A	107.5
C16—C11—C5	120.6 (2)	C32—C31—H31B	107.5
C12—C11—C5	120.8 (2)	C30—C31—H31B	107.5
C11—C12—C13	120.9 (2)	H31A—C31—H31B	107.0
C11—C12—H12	119.5	C32^ii^—C32—C31	117.6 (4)
C13—C12—H12	119.5	C32^ii^—C32—H32A	107.9
C14—C13—C12	119.0 (2)	C31—C32—H32A	107.9
C14—C13—H13	120.5	C32^ii^—C32—H32B	107.9
C12—C13—H13	120.5	C31—C32—H32B	107.9
C13—C14—C15	121.5 (2)	H32A—C32—H32B	107.2
C13—C14—Cl1	119.4 (2)		
			
C4—N1—C1—C10^i^	−176.9 (2)	C4—C5—C11—C16	−82.1 (3)
Fe—N1—C1—C10^i^	−1.4 (3)	C6—C5—C11—C12	−84.1 (3)
C4—N1—C1—C2	0.2 (3)	C4—C5—C11—C12	97.5 (3)
Fe—N1—C1—C2	175.67 (16)	C16—C11—C12—C13	0.1 (3)
N1—C1—C2—C3	−0.3 (3)	C5—C11—C12—C13	−179.5 (2)
C10^i^—C1—C2—C3	176.8 (2)	C11—C12—C13—C14	0.4 (4)
C1—C2—C3—C4	0.3 (3)	C12—C13—C14—C15	−0.3 (4)
C1—N1—C4—C5	179.8 (2)	C12—C13—C14—Cl1	179.66 (18)
Fe—N1—C4—C5	4.3 (3)	C13—C14—C15—C16	−0.4 (4)
C1—N1—C4—C3	0.0 (3)	Cl1—C14—C15—C16	179.65 (19)
Fe—N1—C4—C3	−175.45 (16)	C12—C11—C16—C15	−0.8 (4)
C2—C3—C4—N1	−0.2 (3)	C5—C11—C16—C15	178.7 (2)
C2—C3—C4—C5	−180.0 (2)	C14—C15—C16—C11	1.0 (4)
N1—C4—C5—C6	−1.6 (4)	C1^i^—C10—C17—C22	−72.8 (3)
C3—C4—C5—C6	178.2 (2)	C9—C10—C17—C22	107.5 (3)
N1—C4—C5—C11	176.8 (2)	C1^i^—C10—C17—C18	105.6 (3)
C3—C4—C5—C11	−3.4 (3)	C9—C10—C17—C18	−74.1 (3)
C9—N2—C6—C5	179.4 (2)	C22—C17—C18—C19	−2.3 (4)
Fe—N2—C6—C5	2.9 (3)	C10—C17—C18—C19	179.3 (2)
C9—N2—C6—C7	0.7 (3)	C17—C18—C19—C20	0.4 (4)
Fe—N2—C6—C7	−175.80 (16)	C18—C19—C20—C21	2.0 (4)
C4—C5—C6—N2	−2.2 (4)	C18—C19—C20—Cl2	−178.5 (2)
C11—C5—C6—N2	179.4 (2)	C19—C20—C21—C22	−2.5 (4)
C4—C5—C6—C7	176.3 (2)	Cl2—C20—C21—C22	178.0 (2)
C11—C5—C6—C7	−2.1 (4)	C18—C17—C22—C21	1.8 (4)
N2—C6—C7—C8	−0.6 (3)	C10—C17—C22—C21	−179.8 (2)
C5—C6—C7—C8	−179.3 (2)	C20—C21—C22—C17	0.5 (4)
C6—C7—C8—C9	0.2 (3)	Fe—N3—C23—C24	146.34 (17)
C6—N2—C9—C10	179.6 (2)	N3—C23—C24—C25	104.7 (3)
Fe—N2—C9—C10	−3.9 (4)	N3—C23—C24—C29	−76.1 (3)
C6—N2—C9—C8	−0.6 (3)	C29—C24—C25—C26	−0.4 (4)
Fe—N2—C9—C8	175.94 (16)	C23—C24—C25—C26	178.8 (2)
C7—C8—C9—N2	0.2 (3)	C24—C25—C26—C27	0.2 (4)
C7—C8—C9—C10	−179.9 (2)	C25—C26—C27—C28	0.3 (4)
N2—C9—C10—C1^i^	1.4 (4)	C26—C27—C28—C29	−0.5 (4)
C8—C9—C10—C1^i^	−178.4 (2)	C25—C24—C29—C28	0.1 (4)
N2—C9—C10—C17	−178.9 (2)	C23—C24—C29—C28	−179.1 (2)
C8—C9—C10—C17	1.3 (4)	C27—C28—C29—C24	0.3 (4)
C6—C5—C11—C16	96.4 (3)	C30—C31—C32—C32^ii^	177.2 (4)

Symmetry codes: (i) −*x*+1, −*y*+1, −*z*+1; (ii) −*x*, −*y*, −*z*+2.

### Hydrogen-bond geometry (Å, º)

*Cg*1 and *Cg*7 are the centroids of the N1/C1–C4 and C11–C16 rings, respectively.

**Table:**

*D*—H···*A*	*D*—H	H···*A*	*D*···*A*	*D*—H···*A*
C19—H19···*Cg*1^iii^	0.93	2.66	3.586 (3)	133
C31—H31*A*···*Cg*7^iii^	0.97	2.63	3.701 (5)	160
N3—H3*B*···Cl2^iv^	0.89	2.68	3.651 (2)	133
C7—H7···Cl2^v^	0.93	3.00	3.926 (2)	175

Symmetry codes: (iii) *x*−1, *y*, *z*; (iv) −*x*, −*y*+1, −*z*+1; (v) −*x*, −*y*+1, −*z*+2.
